# Dietary Supplements for Weight Loss and Drug Interactions

**DOI:** 10.3390/ph17121658

**Published:** 2024-12-09

**Authors:** Francisco Rivas García, José Antonio García Sierra, Maria-Isabel Valverde-Merino, Maria Jose Zarzuelo Romero

**Affiliations:** 1 Municipal Health and Consumer Unit, Guadix City Council, 18500 Guadix, Spain; saludyconsumo@guadix.es; 2 Vircell S.L., Health Sciences Technology Park, 18016 Granada, Spain; j.garciasierra0@gmail.com; 3 Pharmaceutical Care Research Group, University of Granada, 18071 Granada, Spain; 4 Department of Pharmacy and Pharmaceutical Technology, Faculty of Pharmacy, University of Granada, 18071 Granada, Spain; mjzarzuelo@ugr.es

**Keywords:** food supplements, drug interactions, obesity

## Abstract

Food supplements are used for a variety of purposes, one of which is weight reduction. As excess weight is a long-term condition, some supplements are expected to be used for long periods of time. The long-term use of these dietary supplements makes it highly likely that they will be combined with medications, increasing the risk of food supplement–drug interactions, which are not always known or disclosed, and can lead to serious health problems, as has been observed. This article discusses some of the compounds used as food supplements for weight reduction (green tea extract, *Garcinia cambogia*, chitosan, quercetin and resveratrol) and the interactions they may cause with some drugs such as: dextromethorphan, buspirone, diclofenac, irinotecan, 5-fluorouracil, cytochrome P450 inducers and inhibitors, statins, orlistat, warfarina, acenocoumarol, fluoxetine, valproate, quetiapine, carbamazepine. This information is expected to be useful for healthcare professionals to detect and intervene on food supplement–drug interactions to ensure the optimization of therapy and patient safety.

## 1. Introduction

Obesity is defined as an abnormal or excessive accumulation of fat in the body that may be detrimental to health, primarily due to an energy imbalance between calories consumed and calories expended [1]. The body mass index (BMI) is often used to assess this. For example, the World Health Organization (WHO) defines overweight as a BMI ≥ 25 kg/m^2^ and obesity as a BMI ≥ 30 kg/m^2^ [1].

Being overweight or obese is a major risk factor for many diseases, including cardiovascular disease (the leading cause of death worldwide in 2019 [2], diabetes, musculoskeletal disorders (especially osteoarthritis), and some cancers (such as endometrial, prostate, kidney, and colorectal cancers) [1].

Overweight and obesity have increased steadily in recent years, to the extent that by 2035, according to the World Obesity Atlas 2023 report, there will be an estimated 4005 million people with a BMI greater than 25 kg/m^2^, representing 51% of the world’s population, of whom 24% will be obese [3]. Epidemic proportions have undoubtedly been reached, with more than 4 million people dying in 2017 as a result of being overweight or obese [4,5]. The size of the dietary supplements market is estimated at USD 139.38 billion in 2024 and is expected to reach USD 173.69 billion by 2029, growing at a CAGR of 4.5% during the forecast period (2024–2029) [3,4,5].

The desire for an esthetically pleasing body and increasing awareness of the risk of developing chronic degenerative diseases means that more and more people are trying to combat this trend [6]. Overweight and obesity are largely avoidable. A balanced diet, limiting energy intake from fats and sugars, and regular physical activity are the most effective ways to reduce the risk of becoming overweight or obese [1]. However, these methods do not produce immediate results and require a strong commitment from the patient [6].

Other approaches to treating obesity include surgery, pharmacological treatment, or the use of dietary supplements [7]. The latter are sometimes preferred by consumers who believe that products of natural origin, unlike synthetic drugs, will not harm their health with negative side effects [6]. Although the efficacy of most dietary supplements for weight control is highly controversial, with conflicting scientific evidence and unclear mechanisms of action, their use is increasing steadily. The quality of these products can sometimes be questionable. Legislation, which is often lax and varies from country to country, and the lack of common definitions may mean that the quality and composition tests to which these products must be subjected are not sufficiently rigorous. Cases have been observed of irregularities in the manufacture of products, incorrect quantities of ingredients contained or intentional or unintentional adulteration with other substances, known or unknown [7,8,9,10,11].

The fact that they are offered as self-care products, are easily accessible (readily available on the Internet) and are promoted as ‘natural’ remedies creates a false perception of harmlessness among the population; this, together with the growing rejection of traditional medicine and the intense advertising that accompanies it, means that sales of food supplements continue to increase. Recent data show that the European market for dietary supplements is estimated at $14.95 billion and is expected to reach $33.80 billion by 2027 [12,13].

Although the sale of these products is not exclusively reserved for pharmacies, the pharmacy is the place where most food supplements (45% of the total) and special weight-loss products (27%) are purchased [8]. The community pharmacist is in a privileged position to provide information on the use and indications of these products, to warn patients of adverse effects, and to avoid possible interactions with the patient’s medication. Providing correct advice to patients about dietary supplements is a real challenge for our profession, as an increasing number of dietary supplements are being marketed, sometimes before solid scientific evidence of their efficacy and safety is available, and the literature on potential drug interactions is constantly being updated. [14].

The coexistence of the two conditions, obesity and dietary supplements, together with the use of medicines for other health problems, justifies the need to be aware of the potential interactions that exist between dietary supplements and medicines and to define the role of the pharmacist in order to avoid, as far as possible, risk situations for the patient. In view of the above, the main objective of this study is to describe the main interactions between medicines and dietary supplements marketed for weight loss.

## 2. Methodology

The work involved a search of the Medline, Cochrane Library, and Scopus databases, structured in three stages:

First—Dietary supplements indicated for weight loss were identified, using the following MeSH terms and Boolean operators: ‘Dietary Supplements’ AND ‘Weight Loss’ OR ‘Anti-obesity Agents’.

Second—Specific ingredients and their mechanisms of anti-obesity action were studied using the following MeSH terms: ‘*Camellia sinensis*’; ‘Catechin’; ‘*Garcinia cambogia*’; ‘Quercetin’; ‘Chitosan’; and ‘Resveratrol’.

Third—Relevant interactions were examined, including in the search equation the Boolean AND operator ‘Drug Interactions’ (Mesh). The subheadings ‘adverse effect’, ‘pharmacokinetics’, and ‘toxicity’ were also included when the search allowed it.

The inclusion criteria, according to which articles were selected, were as follows: (a) articles accessible in full text, written in English or Spanish; (b) articles published within the last five years; (c) articles evaluating interactions between nutritional supplements for obesity and drugs; (d) full text access from any database used; and (e) review articles, clinical trials and randomized controlled trials

Three authors (J-GS, MI-VM, and F-RG) independently identified studies and performed data extraction. To ensure inter-rater agreement, we used percentage agreement, whereby we added the number of times the two reviewers who performed the assessment agreed on the same question, and then divided this sum by the total number of data items considered. To ensure reproducibility and minimize bias, disagreements were resolved by discussion with a third researcher (M-ZR).

The selection was made by reading the title and abstract of the publications, excluding those that clearly did not address the topic of this review and retaining those where there was certainty or doubt. This process included reviewing the title and abstract for screening, then reading other publications, and finally reading the full text of the selected studies. After exhaustive reading of each publication in its entirety, those that definitely did not address the topic of this review were excluded.

For data extraction, a form was used containing the variables of interest: database, journal, authors, article titles, years of publication, languages, countries of origin of the publication, objectives, methods, results, conclusions, and level of evidence.

Initially, according to the defined selection criteria, 390 articles were found, which, after applying the filter of language and age of less than 10 years (except in the case of chitosan, due to the insufficient literature), resulted in 273 records. Of these, 250 were discarded after reading the title, abstract, and/or full text, as they were not related to the specific topic.

**Figure 1:**
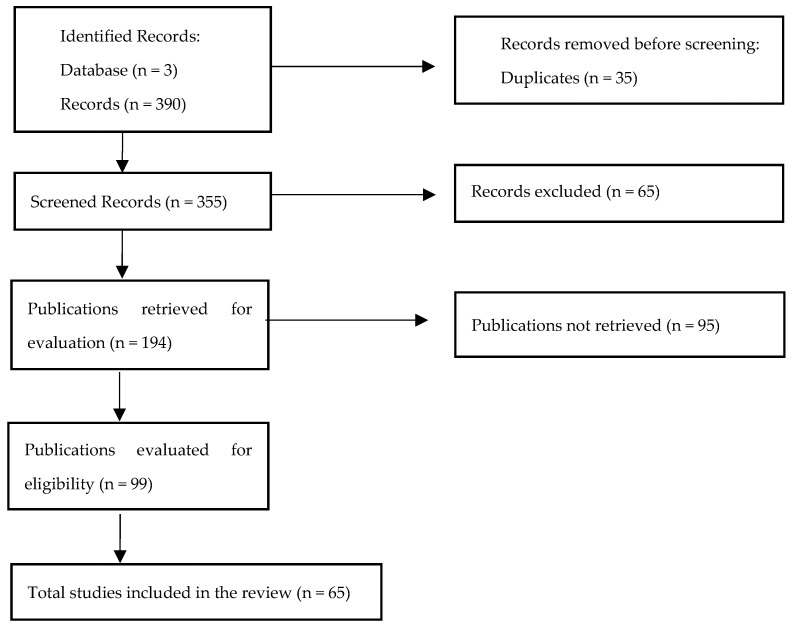
Flow diagram of article selection.

Finally, 27 articles were considered that included interactions of dietary supplements with medicines, 7 that described several weight loss compounds independently (‘Dietary supplements’ [Mesh] AND ‘Anti-Obesity Agents’ [Mesh]), 17 that dealt with mechanisms of action of specific dietary supplements, and 14 included in the sections of introduction and role of the pharmacist. In total 65 articles were included for the review, from which the most relevant information was extracted (Figure 1).

## 3. Results and Discussion

Dietary supplements for weight control act by various mechanisms, such as reducing appetite, modulating energy expenditure and lipid metabolism, or decreasing fat and/or carbohydrate absorption [15].

Most drug–drug interactions occur through a pharmacokinetic mechanism, i.e., supplements interfere with the absorption, distribution, metabolism, or excretion of drugs and may alter their concentration in the blood, which may result in a reduction or exacerbation of their pharmacological effect or an increased risk of adverse effects. Fortunately, most of these interactions, if known, are predictable and the drug dose can be adjusted or the supplement discontinued if necessary [16].

Some of the most commonly used compounds as ingredients in weight loss supplements (green tea extract, *Garcinia cambogia*, chitosan, quercetin, and resveratrol) are discussed below, with an eye to their mechanism of action in obesity and weight loss, as well as possible drug–drug interactions observed in human, animal, and in vitro studies, in order to put what is currently known into context.

### 3.1. Green Tea Extract/Epigallocatechin-3-Gallate (EGCG)

(A) Effect on obesity and weight loss

Green tea is the infusion of the unfermented (unoxidized) leaves of the *Camellia sinensis* plant, which has been used for medicinal purposes throughout history and has been extensively studied in recent decades for its potential health benefits [17]. The properties of green tea are linked to its composition of catechins, a class of flavonoids with potent antioxidant activity, of which epigallocatechin-3-gallate (EGCG) is the most abundant (50–80% concentration) and the most potent antioxidant [18]. Some forms of green tea extract contain low concentrations of caffeine, which is thought to contribute to the anti-obesity effect by suppressing appetite and thermogenesis (increased energy expenditure to produce heat) [19].

Green tea may help to reduce body weight, but the heterogeneity of the studies conducted (in terms of duration, supplement dose, sample size, different ethnicities, caffeine consumption, and participants’ microbiota) means that its efficacy has not yet been confirmed in a quantifiable way [18,19]. Several mechanisms have been proposed to explain this effect; EGCG is thought to alter energy expenditure by inhibiting the enzyme catechol-O-methyltransferase (COMT), thereby delaying the breakdown of norepinephrine (and other catecholamines), which would result in continued stimulation of adrenergic receptors, leading to increased energy expenditure and fat oxidation [17]. In addition, green tea is able to inhibit other enzymes in the gastrointestinal tract, such as pancreatic lipase, amylase, and glucosidase, resulting in reduced absorption of fat and carbohydrates, thereby reducing energy intake [15].

Green tea may also affect the gut microbiota, which may be a secondary mechanism for weight loss through inhibition of amylase and glucosidase, increasing the presence of undigested carbohydrates in the gastrointestinal tract, which bacteria use to produce short-chain fatty acids capable of activating AMP-activated protein kinase (AMPK), thereby reducing lipogenesis and inducing lipolysis. In addition, most tea polyphenols are not absorbed in the small intestine (due to their low bioavailability) and act as a substrate for the gut microbiota [15,17,19,20].

On the other hand, green tea extract and its catechins have been extensively studied for pharmacokinetic interactions. Clinical studies have shown that the maximum concentration (Cmax) and area under the curve (AUC)—parameters that measure the bioavailability of a drug of nadolol, a non-specific beta-blocker—are reduced by 85% in healthy volunteers who were pre-treated with green tea for 14 days before receiving a dose of the drug. Nadolol is a strong substrate of OATP (organic anion transporting polypeptide) transporters found in enterocytes and hepatocytes, specifically OATP1A2. This confirms, with evidence in humans, the decrease in absorption efficiency of EGCG at OATPs that has already been demonstrated in vitro and in animals [18,21]. Reduced bioavailability is also observed with atorvastatin, a lipid-lowering drug that is also a substrate of OATPs [22,23], and with lisinopril, an antihypertensive drug whose transporter is unknown but which, like nadolol, is very hydrophilic. To investigate the effect of catechin on cytochrome P-450 (CYP) isoenzymes, which are responsible for the metabolism of more than 80% of prescription drugs [16], a study was conducted in which patients pre-treated for 4 weeks with EGCG were given a cocktail of different drugs, each a substrate of the major CYP enzymes (caffeine for CYP1A2, dextromethorphan for CYP2D6, losartan for CYP2C9, and buspirone for CYP3A4). No significant changes in enzyme activity were observed. It should be noted, however, that there was a one-day delay between taking the drugs and stopping the green tea extract. This delay may have resulted in the clearance of much of the catechins [18]. Another study, with a smaller number of subjects, duration, and EGCG concentration, concluded that green tea extract consumption did not alter the concentrations of alprazolam and dextromethorphan, which are substrates for the CYP3A4 and CYP2D6 enzymes, respectively [18].

However, in vitro and rodent studies showed the inhibition of a number of CYP enzymes in contact with EGCG. The Cmax and AUC of CYP3A substrates such as diltiazem, verapamil, tamoxifen, simvastatin, and nicardipine were increased in rodents. These drugs are, in turn, transported by the efflux pump P-glycoprotein (P-gp), which has been shown to be inhibited by EGCG in vitro; the effect may be due to inhibition of P-gp rather than CYP3A4. In contrast, concentrations of the CYP1A2 substrate clozapine were reduced due to induction of CYP1A2 by EGCG [18].

Discrepancies between in vitro and in vivo results are not uncommon in drug–drug interaction studies. It has been observed that in vitro CYP2C9 is inhibited by EGCG using diclofenac as a CYP2C9 substrate. However, an in vivo study found that there was no significant difference in Cmax and AUC when fluvastatin (CYP2C9 substrate) was administered in combination with green tea extract compared with fluvastatin alone [24]. One possible explanation for these results is the low membrane permeability of catechins. Catechins must cross membranes to inhibit CYPs (cytosolic enzymes), so the concentration of catechins around CYP2C9 and others after a single dose of green tea extract may not be sufficient to inhibit them. Further studies are needed to clarify whether higher doses of catechins may alter the pharmacokinetics of CYP substrates [24]. On the other hand, a reduction in serum folate was considered when 0.4 mg folic acid was administered with green or black tea (with lower EGCG content). The mechanism of interaction is not defined, but it is thought that tea would interfere with folate absorption in the small intestine [18].

Green tea is used by cancer patients undergoing chemotherapy. One study showed that the AUC of 5-fluorouracil (5-FU) increased by 524% and the Cmax by 151% in the green tea-treated rat group. So, the authors suggested that patients who regularly consume green tea during 5-FU treatment may be candidates for further drug monitoring [25]. The co-administration of EGCG with irinotecan may lead to accumulation of the active metabolite, increasing the risk of adverse reactions [18].

### 3.2. Malabar Tamarind or Garcinia cambogia

(A) Effect on obesity and weight loss

Malabar tamarind, better known by its scientific name *Garcinia cambogia* (GC), is an evergreen tree native to Southeast Asia, India, and Central Africa [26]. The fruits of *G. cambogia* have traditionally been used in cooking and in traditional medicine to treat conditions such as constipation, edema, and irregular menstruation [15,27]. Large amounts of hydroxycitric acid (HCA) are extracted from the exocarp or rind, which is thought to have potential anti-obesity effects [15].

Most GC supplements contain 20–60% HCA [26], which has been shown to be a competitive inhibitor of adenosine triphosphate (ATP)-citrate lyase, thereby reducing the availability of ATP acetyl-Coenzyme A for the formation of fatty acids and cholesterol, attenuating fat accumulation [15]. The anti-obesity effects are also associated with the stimulation of hepatic bglycogenesis, in turn promoting energy expenditure [28] and an increase in satiety through the regulation of serotonin levels [28,29]. However, human studies to test its efficacy in weight reduction have yielded conflicting results. In 20 human studies, 12 reported significant reductions in body weight, and the rest were considered ineffective. It should be mentioned that there are important differences in the design and methodology between the different studies as well as in the type and dose of supplementation administered [28]. Although no adverse effects were found in most of these studies, and the few that did occur were minor (burning, diarrhea, cramps, among others), several cases presenting serious adverse effects have been reported after taking GC (or HCA) supplements in 90 subjects within the dose range recommended by the manufacturers, most of them related to hepatotoxicity (including cases of death), and to a lesser extent cases of toxicity due to serotonin syndrome and psychosis, and cases of rhabdomyolysis [28,30]. In any case, GC consumption should be discouraged in cases such as pregnant women and infants.

HCA can affect the production of fatty acids and cholesterol, which can affect the production of sterols and steroid hormones. Pregnancy is an extremely sensitive period for steroid hormones, so these products are not recommended. On the other hand, patients with depression or manic episodes who take GC should be monitored, because the clinical manifestations of their pathology may worsen [28].

(B) Drug interaction studies

In relation to possible pharmacokinetic interactions of GC, a study was conducted to look at the effect of inhibition on the activity of cytochrome P450 enzymes, which showed that GC extract moderately inhibited CYP2B6 (although this was not so much dependent on HCA, but on other components). It was concluded that an interaction with this enzyme was likely in clinical practice, but there are no in vivo studies to confirm this. In the meantime, caution is advised when consuming GC extract with drugs that are substrates of the CYP2B6 enzyme (such as bupropion, which was used as a substrate in the study) [27].

Very few studies have been conducted on GC or its components, HCA, for drug–drug interactions. However, cases have been reported in which the intake of this food supplement seems to have had an influence on other drugs that patients were already taking. Thus, with regard to hepatotoxicity, one study described the case of a 45-year-old female patient who had been on montelukast for 5 years and who developed liver failure after 7 days of taking 2 food supplements, 1 with GC and the other with C. aurantium. The authors postulated that HCA increased montelukast’s own liver toxicity [30].

Another potentially serious adverse effect associated with GC use is serotonin syndrome and psychosis. HCA has been shown to act as a selective serotonin reuptake inhibitor (SSRI), increasing serotonin levels and serotonin toxicity [28]. This is the case of a 35-year-old woman on stable treatment with escitalopram (an SSRI) for one year, who developed tremors, flushing, and diaphoresis after using a GC extract (60% HCA) supplement for weight loss during the last 2–3 months. She was diagnosed with serotonergic syndrome and treatment with escitalopram was stopped. The patient did not mention the GC intake to her doctor, so after 2 weeks without taking any antidepressant, the doctor prescribed another antidepressant. 

The patient was admitted to hospital with symptoms of serotonin syndrome, and treatment with SSRIs and GC supplements was discontinued [26].

Also, due to cases of rhabdomyolysis [30], it is not recommended to take GC with lipid-lowering drugs such as HMG-CoA reductase inhibitors (statins), as there may be an increased risk of this adverse effect [31]. Given that the efficacy of GC extract has not been fully demonstrated and that the occurrence of serious adverse effects appears to be attributable to HCA, the risk–benefit balance is not in favor of taking this supplement.

### 3.3. Chitosan

(A) Effect on obesity and weight loss

Chitosan is a natural polysaccharide of β-1,4-linked glucosamine residues from the deacetylation of chitin [15]. It is found mainly in the exoskeleton of crustaceans and insects. Although not found naturally in human tissues, chitosan is biodegradable, non-toxic, non-immunogenic and biocompatible, so it is also used in dressings to reduce bleeding [19]. It is important to note that, being derived from crustaceans, chitosan should be avoided in individuals with a shellfish allergy [31].

Oral chitosan appears in many food supplement formulations for the management of obesity, hypercholesterolaemia, and hypertension [7]. This is because it reduces lipid absorption in the gut [19]. This process can be explained by two possible mechanisms: (a) the positive charges of chitosan [32] bind to fatty acids and bile acids (both negatively charged) creating a non-digestible (therefore non-absorbable) complex that is excreted in feces; and (b) the creation of a network effect, by which chitosan could also bind to neutrally charged lipids, such as triglycerides and cholesterol, by hydrophobic interactions [33]. In vitro studies have also suggested that chitosan can modulate adipokine secretion, reducing adipogenesis [31].

Several meta-analyses have attempted to test the efficacy of chitosan in weight loss with conflicting results. Early studies showed significant weight loss, but more recent studies do not show the same results; when only the highest quality clinical trials are analyzed, the average amount of weight lost is only 0.6 kg. Another comparative study between chitosan and orlistat (a pancreatic lipase inhibitor) concluded that chitosan did not inhibit the absorption of dietary fat [7].

(B) Drug interaction studies

Chitosan may scavenge fat-soluble substances, thereby interfering with the absorption of vitamins A, D, E, and K (fat-soluble) [31], which in combination with vitamin K antagonists such as warfarin and acenocoumarol may increase the anticoagulant effect, thereby increasing the risk of bleeding. In addition, it has been observed that chitosan may interfere with the coagulation cascade process [20].

It was concluded that chitosan was able to sequester fluoxetine and vitamin B12. Although they point out that at recommended doses the interaction is unlikely to be clinically relevant, they do warn that it may be a problem when there is excessive consumption of chitosan. This justifies the need for further studies with other substances that may interact with chitosan in a similar way [31,32,33,34]. No in vivo studies have been found to confirm this interaction. However, two cases were reported for a probable interaction between chitosan and sodium valproate, a commonly used anticonvulsant, presumably by this or a similar interaction mechanism. Both cases [35] involved young women on stable treatment with valproate and free of seizures who, after a few days of taking chitosan as a food supplement, experienced seizure symptoms. The plasma concentration of valproate was undetectable and the seizures stopped when the chitosan was removed. It is thought that the negative side of the carboxylic group of valproate may have bound to the positive side of chitosan and prevented its absorption, resulting in the drop in blood concentration.

Although chitosan may have shown promising results, the reality is that studies to date have not been able to demonstrate its efficacy in the management of obesity, and the adverse effects, however few and however mild (bloating, constipation, nausea, etc.), mean that, for the time being, chitosan cannot be recommended for weight reduction [7,15,31].

### 3.4. Quercetin

(A) Effect on obesity and weight loss

Quercetin is one of the most abundant flavonoids in fruits and vegetables and one of the most widely consumed in the daily diet; it is mainly found in onions, apples, grapes, and beverages such as white tea [36,37]. Quercetin is thought to have antioxidant, anti-diabetic, anti-inflammatory, and anti-obesity properties [38].

Quercetin is thought to help reduce obesity through several mechanisms. These effects have been studied in cell culture and in vitro, but human studies are very limited. One of the first observations in rodents was that quercetin binds directly to the glucose transporter GLUT4 (overexpression of which is associated with obesity), inhibiting glucose uptake [39]. Quercetin may also be able to reduce obesity in rodents on a high-fat diet by modifying the gut microbiota; however, it appears that most of the anti-obesity effects of quercetin are due to its antioxidant and anti-inflammatory properties [37,38,39].

Obesity is the result of an imbalance between calorie intake and lack of energy expenditure, which leads to a constant expansion of adipose tissue, causing hypertrophy and hyperplasia, resulting in increased permeability of adipose tissue to macrophages. The lack of oxygen causes macrophages to become activated and produce pro-inflammatory cytokines. Elevated levels of these, including TNF-α, IL-6, and IL-1β (which have been shown to be over-expressed in obese people), cause a state of chronic low-intensity inflammation in obese people. In addition, high levels of these can affect systemic insulin sensitivity and promote the development of cancer [38].

Quercetin has been shown in in vitro and animal studies to reduce the levels of over-expressed pro-inflammatory molecules in obese people by altering the expression of their genes [38,40] through the inhibition of several signaling pathways [38]. In addition, quercetin treatment has also been shown to reduce the expression of peroxisome proliferator-activated receptor γ (PPARγ), the key regulator of adipocyte differentiation, suggesting that quercetin may thus reduce adipogenesis [41]. Several studies in rodents have confirmed weight loss with quercetin in treatments lasting at least two weeks [41,42], as well as improved adipocyte hypertrophy and reduced lipid accumulation [38]. At the same time, a reduction in blood glucose levels has been observed with short treatments, which may improve the management of diabetes [41]. This may be due to a reduction in oxidative stress and attenuation of β-pancreatic cell damage [42]. There are insufficient human trials to suggest that quercetin may reduce BMI, waist circumference, and triglyceride concentrations in overweight and obese people [42]; however, other trials have shown no significant differences compared with placebo [39].

(B) Drug interaction studies

Various studies have demonstrated a significant inhibition by quercetin of several isoenzymes of cytochrome P-450. An increase in Cmax and AUC of chlorzoxazone, a substrate of CYP2E1, was observed; this enzyme also catalyzes the metabolism of ethanol [43] which could minimize the hepatotoxicity of alcohol. Diclofenac also showed an increased plasma concentration after the intake of quercetin. The enzyme CYP2C9 catalyzes the transformation of diclofenac to its pharmacologically more active metabolite, 4′-hydroxy-diclofenac, whose bioavailability was reduced with the administration of quercetin [44]. Therefore, it is assumed that the combination of quercetin and diclofenac is not recommended for pain treatment [36].

In vivo studies in rodents have also observed a significant increase in the concentration of warfarin, metabolized by CYP2C9, and quetiapine in rats pretreated with quercetin. The latter suggests that the increased plasma concentration of quetiapine may be due, in addition to the inhibition of CYPs, to the inhibition of P-glycoprotein (P-gp), leading to a reduction in the permeability of the blood–brain barrier (BBB), preventing quetiapine from acting in the central nervous system and further increasing the amount of drug in plasma. The authors indicate a potentially relevant interaction [45,46].

In vitro studies have shown that quercetin is a potent inhibitor of CYP1A2 [47] and CYP3A4, an effect confirmed in rodents [48]. For CYP1A2, the doses of drugs metabolized by this enzyme (caffeine, melatonin, and clozapine) should be monitored [49]. Quercetin also increased in several studies in rats the concentration of substrates of CYP3A4 (doxorubicin, valsartan, cyclosporine, tamoxifen, and pioglitazone) [50].

In addition to its role in overweight and obesity, quercetin is widely used in cancer patients. Tucatinib, a tyrosine kinase inhibitor used in the treatment of HER-2 positive cancers, is primarily metabolized by CYP2C8; in rats, it has been observed that co-administration with quercetin significantly increased its bioavailability. An in vitro study conducted with palbociclib and ribociclib, selective inhibitors of cyclin-dependent kinases 4 and 6 (CDK-4/6), demonstrated that in both cases, the half-life was significantly prolonged, depending on the concentration of quercetin. It is noted that the pharmacokinetic changes observed may also occur in humans [46,51]. Therefore, co-administration of quercetin with tucatinib, palbociclib, or ribociclib should be avoided to prevent the occurrence of adverse effects.

Finally, quercetin has been shown to bind to human serum albumin with high affinity in in vitro studies. Moreover, it is capable of displacing molecules already bound to albumin. This is the case with amlodipine, an antihypertensive drug that shares an active binding site on albumin, although with less affinity than quercetin, which displaces it. This causes the free fraction of amlodipine (responsible for the pharmacological effect) to increase in blood, enhancing its effect and potentially altering the patient’s blood pressure, thereby increasing the risk of adverse reactions [52].

### 3.5. Resveratrol

(A) Effect on obesity and weight loss

Resveratrol is a natural polyphenol, specifically a stilbene, primarily found in red grapes, berries, and medicinal plants such as Polygonum cuspidatum. Resveratrol was considered the source of the “French paradox,” a phenomenon in which the French population, despite having a diet rich in fats, had a lower prevalence of obesity and cardiovascular diseases; this was attributed to red wine, which has a high content of this polyphenol [53]. Resveratrol is credited with a series of antioxidant, anti-inflammatory, cardioprotective, and anti-obesity properties.

In vitro and animal studies have found that resveratrol improves the metabolic syndrome profile and prevents weight gain. Additionally, it reduces adipogenesis and the viability of pre-adipocytes. However, its effect in humans appears to be much more moderate; although weight reductions have been observed compared to control groups, these are often not clinically significant [15]. Resveratrol exerts its anti-obesity effects through various mechanisms that are not yet fully understood.

It should be noted that resveratrol is a potent activator of NAD-dependent sirtuin-1 deacetylase (SIRT-1), an enzyme that plays a key role in aging and metabolic stress processes and is implicated in glucose tolerance, obesity development, and cancer. This enzyme activates signaling pathways resulting in the inhibition of adipogenesis and adipocyte differentiation. Additionally, SIRT-1 activates PGC1α (Peroxisome proliferator-activated receptor γ co-activator 1 α), leading to increased mitochondrial activity, gluconeogenesis, and the inhibition of glycolysis in the liver. SIRT-1 levels are higher in individuals subjected to calorie-restricted diets and physical exercise, suggesting that resveratrol may mimic similar activation pathways of validated therapies against obesity [53]. It also promotes the phosphorylation of AMPK (which typically occurs in states of energy deficiency). Dephosphorylated AMPK is a key signal for the induction of adipogenesis, so its phosphorylation hinders this process. In vitro, a reduction in adipogenesis was also observed due to lower expression of genes that favor it, such as PPARγ [54].

Resveratrol also acts on brown adipose tissue (BAT). BAT, which decreases with aging, functions to maintain body temperature by transforming energy into heat (thermogenesis). The capacity of BAT to dissipate energy is due to the increased number of mitochondria in brown adipocytes, which contain high amounts of uncoupling protein 1 (UCP1), expressed exclusively in them [54]. Additionally, studies in rodents have shown that resveratrol increases UCP1, SIRT-1, and AMPK levels in BAT, thereby increasing energy expenditure. Furthermore, in mice, an induction of specific BAT genes has been observed, suggesting that resveratrol could induce the conversion of white adipose tissue to brown (“browning”) [54,55,56].

(B) Drug interaction studies

Resveratrol has been shown to be a potent inhibitor of the CYP3A4/5 enzyme, strongly inhibiting the metabolism of testosterone and midazolam (substrates of this enzyme). This phenomenon occurs irreversibly through a non-competitive mechanism at micromolar concentrations of resveratrol. Such concentrations can be reached after a dose of one gram of resveratrol, which is not typically attained in a daily diet. However, some resveratrol dietary supplement formulations can reach concentrations of 2–5 g per day, where interaction with a drug substrate of the CYP3A4/5 enzyme is likely [57].

To confirm that the in vitro interactions observed could occur significantly in humans, Bebeda et al. [58] conducted trials on 12 healthy adults, observing the effects of administering substrates of different CYP enzymes in patients pretreated with resveratrol. They found that carbamazepine (CBZ), metabolized in the liver to its pharmacologically active form (10,11-epoxide of carbamazepine, CBZE), showed increased Cmax and AUC values in patients treated with resveratrol, resulting in a significantly reduced CBZE/CBZ ratio, which could compromise epilepsy management and increase the risk of adverse effects from CBZ. This provides in vivo evidence of the inhibition of the CYP3A4 enzyme. Carbamazepine, in addition to having a narrow therapeutic margin, is a commonly used drug in long-term antiepileptic regimens, making the risk of interaction high in patients using both substances concomitantly [58].

On the other hand, resveratrol has also shown inhibitory activities on the enzymes CYP2C9 and CYP2E1. Similarly, an increase in the bioavailability (Cmax and AUC) of diclofenac [59] and chlorzoxazone [60] was confirmed, indicating the inhibition of CYP2C9 and CYP2E1, respectively, in healthy patients pretreated with resveratrol. These findings draw similar conclusions to those of quercetin, suggesting a possible modification of the effects of drugs metabolized by CYP2C9 (such as losartan) and aiding in the prevention of ethanol-related hepatotoxicity.

Resveratrol has also demonstrated in voluntary subjects that it can inhibit CYP1A2, modifying caffeine concentration, and CYP2D6, affecting dextromethorphan levels. Finally, the potent inhibition of CYP2C19, a substrate of omeprazole, has also been observed in vitro [57,58,59,60,61].

### 3.6. Other Compounds of Interest

There are other ingredients in dietary supplements for weight reduction of interest due to their possible interactions:

- Conjugated Linoleic Acid (CLA) is a fatty acid with a conjugated double bond, abundant in oils, and to a lesser extent, in the meat and milk of ruminants, although it is mass-produced from linoleic acid. In animals, significant reductions in total body weight have been observed due to increased lipolysis, fatty acid oxidation, and reduced lipogenesis (through changes in PPAR gene expression) [62]. It also promotes “browning”, increasing thermogenesis [63]. Despite this, the results have not been extrapolated to humans. On the other hand, CLA has been shown to increase, through unknown mechanisms, tissue levels of retinol (vitamin A alcohol) and its sole specific circulating carrier protein, retinol-binding protein (RBP), which means these two molecules may compete to be substrates in various metabolic pathways [64].
- Glucomannan is a soluble fiber capable of absorbing 50 times its weight in water, thus increasing gastric emptying time and the sensation of fullness. Glucomannan cannot be digested until it reaches the colon, where it is fermented by the microbiota. A meta-analysis of studies conducted in humans found no significant differences in weight reduction when compared to a placebo [65].
- Green coffee bean extract, chromium picolinate, and curcumin are other compounds that could potentially have anti-obesity properties and are used as dietary supplements. Limited scientific evidence has been found, and there is practically no literature addressing drug interactions [15].

## 4. Conclusions

The increase in overweight and obesity in today’s society suggests that there will be more patients using dietary supplements for weight reduction.

The fact that the majority of the population perceives them as completely safe encourages their widespread use among people with chronic diseases.

The long-term use of these dietary supplements makes it highly likely that they will be combined with medications, increasing the risk of food supplement–drug interactions, which are not always known or disclosed, and can lead to serious health effects, as has been observed.

Understanding and evaluating the interactions that dietary supplements can have with medications is vital to prevent their occurrence and to avoid exacerbating health problems.

Healthcare professionals, including physicians and pharmacists, have an important role to play in the identification, analysis, and prevention of food–drug interactions in order to ensure adequate optimization of drug treatment and reduce healthcare costs associated with morbidity and mortality.

To this end, different strategies need to be implemented at the research and clinical level, such as the following:

- Identify and analyze the different interactions observed during the research and design of new medicines, for their inclusion in the technical data sheets and package leaflets.
- In clinical trials, an interaction risk assessment should be carried out, as well as monitoring of the formulation development procedure.
- In usual healthcare practice, incorporate strategies for the detection and intervention of possible interactions, within the framework of the pharmacotherapeutic follow-up carried out by the pharmacy.
- Promote knowledge and access to databases, on which to base health interventions for patients at risk or affected by an interaction between nutritional supplements and drugs can be based.
